# Sparse Spatial Scaffolding for Visual Working Memory

**DOI:** 10.1523/JNEUROSCI.0318-25.2026

**Published:** 2026-03-31

**Authors:** Baiwei Liu, Zampeta-Sofia Alexopoulou, Siyang Kong, Anne Zonneveld, Freek van Ede

**Affiliations:** Institute for Brain and Behavior Amsterdam, Department of Experimental and Applied Psychology, Vrije Universiteit Amsterdam, Amsterdam 1081 HV, The Netherlands

**Keywords:** eye movement, internal attention, oculomotor system, spatial coding, visual working memory

## Abstract

When holding information “in mind,” it is vital to keep individual representations separated and selectively accessible for guiding behavior. Space is known to serve as a foundational scaffold for mnemonic individuation, yet the format and flexibility of spatial scaffolding for working memory remain elusive. We hypothesized that information in working memory can be recoded from its native format at encoding to organize and retain internal representations sparsely. To test this, we presented to-be-memorized visual items at distinct directions and distances and leveraged gaze biases during mnemonic selection as an implicit read-out of spatial scaffolding for working memory. We report how male and female humans abstract away over incidental item distance when direction alone suffices as a scaffold but incorporate distance when it aids mnemonic individuation. This suggests the flexible use of a sparse spatial scaffold for working memory, resorting to the minimal spatial scaffold required for the individuation of internal representations.

## Significance Statement

A foundational task faced by the brain is to not only maintain relevant information “in mind” but also to keep maintained representations separate from each other and selectively accessible for guiding behavior. It has long been known that spatial configurations serve as a foundational scaffold for working-memory organization, but the nature and flexibility of such spatial scaffolding have remained unclear. By probing biases in fixational gaze behavior during mnemonic selection, we could uniquely uncover spatial coding for working memory through the eyes—without ever asking about memory for location. Doing so, we uncover the flexible nature of spatial scaffolding for memory: retaining those spatial properties that aid memory organization while abstracting away over spatial properties that are obsolete.

## Introduction

A central challenge for working memory is to code for information in a format in which individual representations remain separated and can be selectively prioritized for guiding behavior (Baddeley, 1992; Kahneman et al., 1992; Treisman, 1996; Luck and Vogel, 1997; D”Esposito and Postle, 2015; van Ede and Nobre, 2023). Within the study of visual working memory, ample studies have made clear how space serves as a foundational scaffold for the separation and selection of individual working-memory representations (Treisman and Zhang, 2006; Hollingworth, 2007; Kuo et al., 2009; Theeuwes et al., 2011; Abrahamse et al., 2014; Pertzov and Husain, 2014; Foster et al., 2017; Manohar et al., 2017; Schneegans and Bays, 2017; de Vries and van Ede, 2024). Yet, how precisely space is used for working memory remains elusive and has been the subject of ample recent investigation.

Several recent studies have made clear how, once visual information has been encoded into working memory, the use of spatial location as a scaffold for memory individuation and selection is not necessarily veridical but can be flexibly updated. For example, spatial organization in visual working memory can be transformed (Ester et al., 2009; Woodman et al., 2012; Williams et al., 2013; Brincat et al., 2021; Chernik et al., 2024; Mössing et al., 2024; Liu et al., 2024a), can be compressed (Willeke et al., 2022), and engages additional spatial frames of reference (Aagten-Murphy and Bays, 2019; Draschkow et al., 2022; Liu et al., 2024a). This flexible nature of working memory—in which information can be recoded from its native format at encoding (see also Rainer et al., 1999; Brady et al., 2009; Woodman et al., 2012; Lee et al., 2013; Boettcher et al., 2021; Henderson et al., 2022; Kwak and Curtis, 2022; Kandemir et al., 2024)—provides the opportunity to tune the spatial scaffolding for working memory to the task in an adaptive and efficient manner. Accordingly, working memory may spatially organize and retain mnemonic visual contents sparsely: utilizing more sparse (abstracted) spatial scaffolds as long as they suffice for mnemonic individuation and selection and engaging richer (more veridical) spatial scaffolds only when aiding mnemonic individuation.

In the current study, we leveraged spatial biases in fixational gaze behavior to assess the hypothesis of sparse spatial scaffolding for visual working memory. Research has shown that spatial biases in fixational gaze behavior can reflect the spatial allocation of attention (Hafed and Clark, 2002; Engbert and Kliegl, 2003; Martinez-Conde et al., 2004; Rolfs, 2009; Rucci and Poletti, 2015), including when selecting memorized visual information held in working memory (van Ede et al., 2019; Draschkow et al., 2022; Chawoush et al., 2023; de Vries et al., 2023; Liu et al., 2024a,b; Wang and van Ede, 2025). To tackle our current hypothesis, we presented visual memory items at different directions and distances from fixation such that item distance was either useful or redundant as a spatial scaffolding feature for individuating memory contents. Critically, by studying spatial biases in fixational gaze behavior during mnemonic selection, we could gain insights into the spatial scaffold that was implicitly used for working memory—that is, without us ever explicitly asking participants about memorized item locations.

Our results reveal the phenomenon of flexible and sparse spatial scaffolding for working memory: showing the use of fundamentally different spatial codes depending on the spatial layout of memory. Specifically, we show how humans rely primarily on memorized item direction—regardless of item distance—when direction is sufficient for mnemonic individuation and additionally utilize distance when direction alone is insufficient as a scaffold because multiple items in working memory share the same direction.

## Materials and Methods

### Ethics

Experimental procedures were reviewed and approved by the local Ethics Committee of the Vrije Universiteit Amsterdam. Each participant provided written consent before participation and was reimbursed €10/h.

### Participants

We conducted three experiments with independent participant recruitment. Twenty-five healthy human volunteers participated in each experiment (Experiment 1: age range, 18–31; 6 male and 19 female; 23 right-handed and 2 left-handed; Experiment 2: age range, 18–27; 3 male, 21 female, and 1 non-binary; 24 right-handed and 1 left-handed; Experiment 3: 20–30; 7 male and 18 female; 22 right-handed and 3 left-handed). Sample size of 25 per experiment was set a priori based on previous publications from our lab that relied on the same outcome measure (van Ede et al., 2019; 2020; Draschkow et al., 2022). In Experiment 3, to achieve the intended sample size, two participants were replaced due to the poor quality of the eye-tracking data. No participants required replacement in Experiments 1 and 2. All participants had normal or corrected-to-normal vision.

### Stimuli and procedure

We designed a visual working-memory task in which we never tested the location of specific memory items, but in which space served as a scaffold for item separation and selection. In the critical versions of our tasks, we presented visual memory items at different directions (left/right/top/bottom) and distances (3 or 6°) from fixation and manipulated the spatial layout such that item distance was either useful as a spatial scaffolding feature (because direction alone was insufficient) or redundant (because direction alone was sufficient). Below, we first explain the basic task, before returning to our two key manipulations.

**Figure 1.**
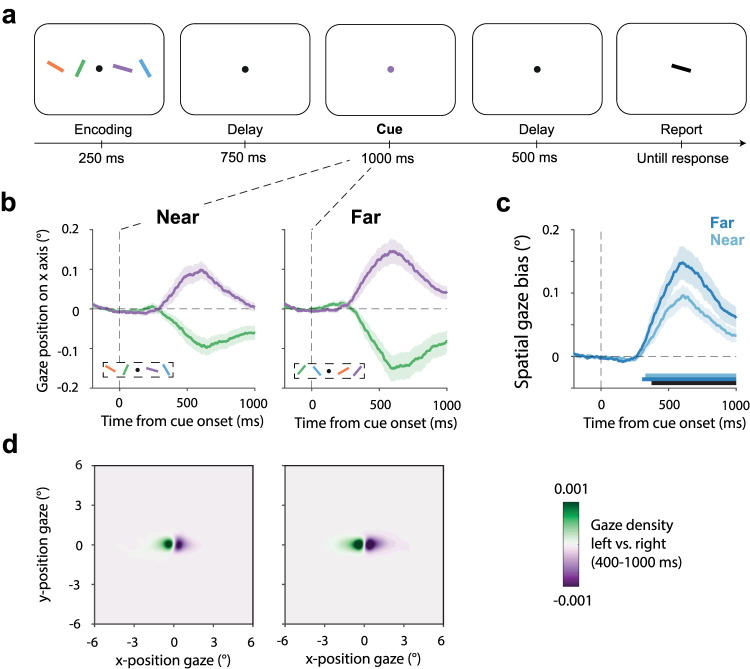
Fixational gaze behavior during mnemonic selection tracks the use of both direction and distance as spatial scaffolding features for visual working memory. ***a***, Task schematic. Participants memorized four bars with different colors and orientations. Following a delay, a color change of the central fixation dot (retro-cue) prompted participants to select the color-matching item from working memory to compare its orientation to a black bar in the upcoming test display. Memory items were presented to the left or right and near (3°) or far (6°) from fixation at encoding, but participants were never asked about memorized item location. ***b***, Time course of average gaze position following central retro-cues that prompted the selection of the memory item that had been presented to the left or right at encoding, separately for when the memory item was near (left panel) or far (right panel) at encoding. ***c***, Time courses of spatial biases in gaze toward the cued memory item when this item was near (light) or far (dark) at encoding. ***d***, Heat maps of two-dimensional gaze position following left versus right cues. Shading in panels ***b*** and ***c*** indicate ±1 SEM calculated across participants (*n* = 25).

In all three experiments, participants engaged in a visual working-memory task that required the selection of a visual item from working memory for an upcoming orientation comparison (Fig. 1*a*). Though we manipulated spatial features of the display, note how we never asked participants about memorized item direction, nor about memorized distance. To perform our task, memories of color-orientation bindings were sufficient, in principle. Spatial location merely served as a scaffold for separating and selecting specific memory items.

Each trial began with a start fixation (200 ms) followed by a brief (250 ms) encoding display where four bars with different colors and orientations appeared on either two or four directions of the fixation dot. After a 750 ms working-memory retention interval, the fixation dot changed color for 1,000 ms serving as a retro-cue. This retro-cue cued with 100% validity which memory item (i.e., the color-matching item) would have to be compared with the upcoming test stimulus. The retro-cue was followed by another retention delay of 500 ms before the test display appeared. During the test display, a black bar appeared at the center of the screen. The test bar was always rotated between 10 and 20° clockwise or counterclockwise from the cued bar in memory. Participants reported whether the cued memory item should be turned clockwise or counterclockwise to match the black bar in the test display. Participants received feedback immediately after response by a number (“0” for wrong or “1” for correct) appearing for 250 ms slightly above the fixation dot. After the feedback, intertrial intervals were randomly drawn between 500 and 1,000 ms.

**Figure 2.**
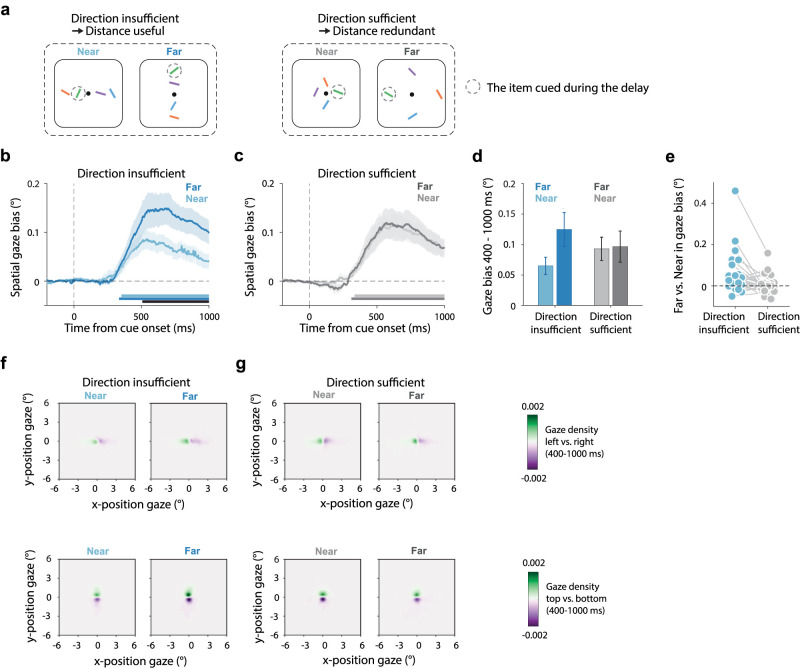
Fixational gaze behavior reveals a sparse spatial scaffold that abstracts over distance when direction alone is sufficient for mnemonic individuation. ***a***, Representative encoding displays used in Experiment 2. In Experiment 2 we additionally manipulated the usefulness of distance as a scaffolding feature for mnemonic individuation. The “direction-insufficient → distance-useful” condition (left) mirrored Experiment 1 where direction alone was insufficient for mnemonic individuating, rendering distance useful as an additional scaffolding feature. In the alternative “direction-sufficient → distance-redundant” condition (right), the four items each had a unique direction, rendering direction sufficient for item individuation and distance redundant as an additional scaffolding feature. Dashed circles indicate the later cued item in the different conditions (dashed circles were never shown in the experiment). ***b***, ***c***, Time courses of spatial biases in gaze toward the cued memory item when this item was near (light) or far (dark) at encoding, when direction is insufficient and distance useful as a spatial scaffold (panel ***b***) or when direction was sufficient and distance redundant as an additional spatial scaffolding feature (panel ***c***). ***d***, Gaze bias averaged across the a priori defined window from 400 to 1,000 ms after the cue (based on van Ede et al., 2019). ***e***, Individual data for the far versus near comparison in the direction-insufficient and the direction-sufficient conditions. ***f–g***, Heat maps of two-dimensional gaze position following left versus right cues (top row) or top versus bottom cues (bottom row) across our four conditions. Shading in panels ***b*** and ***c*** and error bars in panel ***d*** indicate ±1 SEM calculated across participants (*n* = 25).

**Figure 3.**
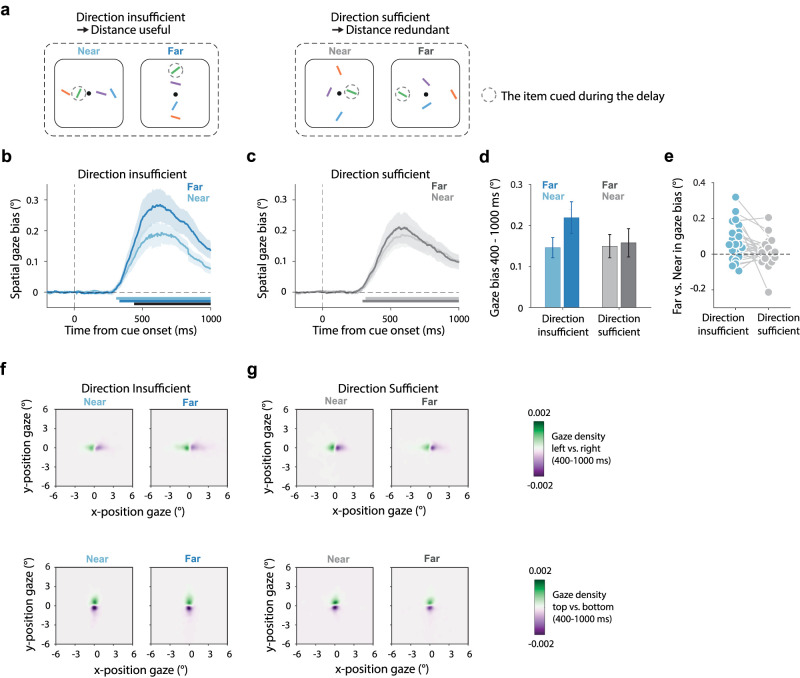
Our findings are driven by spatial scaffolding demands, not the mere presence of near and far items. ***a***, Representative encoding displays used in Experiment 3. Experiment 3 replicated Experiment 2 except that in Experiment 3, the “direction-sufficient → distance-redundant” condition (right-top panel) now always also had two near items (on one axis) and two far (on the other axis). ***b***, ***c***, Time courses of spatial biases in gaze toward the cued memory item when this item was near (light) or far (dark) at encoding, when direction was insufficient and distance useful as a spatial scaffold (panel ***b***) or when direction was sufficient and distance redundant as an additional spatial scaffolding feature (panel ***c***). ***d***, Gaze bias averaged across the a priori defined window from 400 to 1,000 ms after the cue (based on van Ede et al., 2019). ***e***, Individual data for the far versus near comparison in the direction-insufficient and the direction-sufficient conditions. ***f–g***, Heat maps of two-dimensional gaze position following left versus right cues (top row) or top versus bottom cues (bottom row) across our four conditions. Shading in panels ***b*** and ***c*** and error bars in panel ***d*** indicate ±1 SEM calculated across participants (*n* = 25).

Our key experiments (Experiments 2 and 3) had two key manipulations (Figs. 2*a*, 3*a*). We manipulated the following: (1) whether cued memory items were presented near (3°) or far (6°) from fixation, and (2) whether all items were presented in a unique direction (left/right/bottom/top) or whether certain directions were shared between multiple items in the array (two left and two right items or two top and two bottom items). Critically, in the first condition (Fig. 2*a*, labeled “direction-insufficient → distance-useful”), there were always two items competing along each direction of an axis (e.g., two up and two down or two left and two right), rendering direction alone insufficient as a spatial scaffold for individuating the four items and rendering distance useful as an additional scaffold. In contrast, in the alternative condition (Fig. 2*a*, labeled “direction-sufficient → distance-redundant”), all items were associated with a unique direction, such that direction was sufficient as a spatial scaffold for individuating the four memory items, rendering distance redundant.

Experiment 1 (Fig. 1*a*) only included the condition with four items on a line (direction-insufficient → distance-useful) and served to validate the sensitivity of our spatial marker to track the use of both “direction” and “distance” when both features were useful as a scaffold. In Experiments 2 and 3, we added the direction-sufficient (distance-redundant) condition in which all items were presented in a different direction from fixation (Figs. 2*a*, 3*a*). In Experiment 2 (Fig. 2*a*), in this direction-sufficient (distance-redundant) condition, the display in a given trial always contained four items that were either all near (3°) or all far (6°). In contrast, in Experiment 3 (Fig. 3*a*), displays in this condition always contained two near items (one axis) and two far items (on the orthogonal axis), to equate the total number of near and far items in each display between the two conditions in which direction was either sufficient (and distance redundant) or insufficient (and distance useful) as a spatial scaffold for item individuation. Please note that in Experiment 1, we only included the horizontal configuration (as depicted in Fig. 1*a*), while in Experiments 2 and 3, we included both vertical and horizontal configurations in the direction-insufficient (distance-useful) condition (as depicted in Figs. 2*a*, 3*a*), such that we equally often cued a left/right/top/bottom item in both the direction-insufficient (distance-useful) and direction-sufficient (distance-redundant) conditions.

In the encoding display, bars were randomly assigned two or four distinct colors from the color pool: green (RGB: 133, 194, 18), purple (RGB: 197, 21, 234), orange (RGB: 234, 74, 21), and blue (RGB: 21, 165, 234). Bars were also drawn at distinct orientations ranging from 0 to 180° with a minimum difference of 20° between each other. During the test display, the bars were always dark gray (RGB: 64, 64, 64) and were always oriented clockwise or counterclockwise from the cued memory target, with a change in orientation randomly drawn between 10 and 20°. The bar is 2 visual degrees in length and 0.4 visual degrees in width and the fixation point has a radius of 0.07 visual degrees.

Experiment 1 consisted of four sessions that each contained 10 blocks of 16 trials, resulting in a total of 640 trials. In Experiments 2 and 3, we added the direction-sufficient (distance-redundant) condition and therefore increased the number of trials. Experiments 2 and 3 each consisted of five sessions, where each contained five blocks of 32 trials, resulting in a total of 800 trials. Conditions were randomly mixed within each block.

Before the start of each experiment, participants practiced the task for 5 min. Experiment 1 required ∼80 min while Experiments 2 and 3 each required ∼100 min per participant to complete. Participants were instructed to keep fixation throughout the task, but trials were not aborted when larger eye movements occurred. The encoding display was too brief to allow gaze fixations to all memory items, and during the main cue-period of interest there was nothing to look at on the screen apart from the central fixation dot.

### Eye tracking

We used an EyeLink 1000 (SR Research, with 1,000 Hz sampling rate) to track gaze from a single eye (right eye in all participants except 1 for which the left eye provided a better signal). The eye-tracker camera was positioned on the table ∼5 cm in front of the monitor and ∼65 cm in front of the eyes.

Gaze data were read into Matlab using the FieldTrip toolbox (Oostenveld et al., 2011). In line with previously established protocols (van Ede et al., 2019; 2020; Liu et al., 2022), we first identified blinks and replaced identified blink clusters (extending 100 ms before and after detected blinks) with Not-a-Number (NaN) to effectively mitigate any artifacts from the blinks. Then, data were epoched from −1,000 to +2,000 ms relative to the onset of the retro-cue.

We focused our analysis on spatial biases in gaze position over time in response to the central color retro-cue that prompted the selection of one out of the four items in working memory. Because the cue was nonspatial and the test stimulus would occur centrally, any spatial bias in gaze during this period must reflect selection within the spatial layout of visual working memory.

To investigate directional biasing of gaze during mnemonic selection, we first baseline corrected the eye data by subtracting the mean data in the [−200 to 0 ms] baseline window. Then, we examined eye data on the *x*-axis when the cued item was on the left or the right side, on the *y*-axis when the cued item was above or below fixation. Following our prior studies (van Ede et al., 2019; 2020; Draschkow et al., 2022), we condensed the relevant data from the left/right and top/bottom trials into a single gaze time course of “towardness” that captured the bias of gaze toward the memorized location of the cued memory item. We could then compare this measure of towardness across our experimental conditions.

To zoom in on biases in fixational gaze behavior, we removed occasional trials with large gaze deviations from fixation (as was also done in van Ede et al., 2020; 2021; de Vries et al., 2023) within the period of interest from 0 to 1,000 ms relative to cue onset. Specifically, we removed trials in which gaze values were larger than 2° away from fixation, which corresponded to the inner radius of the near item positions. This ensured that all reported effects cannot be driven by looking at the original location of the cued memory items, but instead are fixational in nature. Gaze remained close to fixation in the majority of trials; hence, relatively little trials were removed following this procedure: (usable trials: Experiment 1: 95 ± 1.4%, Experiment 2: 93 ± 1.7%, Experiment 3: 91 ± 2.6%).

In addition, the fixational nature of the gaze bias was also visualized through two-dimensional (2D) heat maps of gaze density (as in van Ede et al., 2019; 2020; Draschkow et al., 2022). For this, we collated gaze-position values across time and trials (without averaging) using the data from the 400–1,000 ms window after the retro-cue (a time window set a priori based on van Ede et al., 2019). We then counted the number of gaze samples within 0.1° × 0.1° bins, ranging from −6 to 6° and sampling the full 2D space in steps of 0.05°× 0.05°. To obtain a density map, we divided 2D gaze-position counts by the total number of gaze-position samples. For visualization purpose, density maps were smoothed using a 2D Gaussian kernel with an SD of 0.25° × 0.25° (using the built-in function “imgaussfilt” in MATLAB). To provide a comprehensive and undistorted view of gaze density in our task, trials with large gaze deviations were not removed from the data entering these gaze-density visualizations.

To represent the heat map associated with the directional gaze biases of interest, we first obtained the maps separately following cues to left, right, top, and bottom items and subtracted maps between conditions in which cues were associated with items in the opposite direction: left versus right and top versus bottom. We did this separately for each of our core experimental conditions.

To assess whether differences in behavioral performance between our direction-sufficient and direction-insufficient conditions could account for the observed gaze-bias effects, we quantified a gaze-bias interaction index, defined as the difference between the far–near gaze bias in the direction-insufficient condition minus the corresponding far–near gaze bias in the direction-sufficient condition. We then computed the correlation between this gaze-bias interaction index and the difference in accuracy between the two experimental conditions across participants. To maximize sensitivity, we pooled the data from Experiments 2 and 3 for this additional analysis.

### Complementary microsaccade analysis

To supplement our primary analyses that focused on biases in gaze position, we also ran an analysis of fixational saccades (microsaccades). Saccades were detected using a velocity-based detection algorithm as described and validated in our previous work (Liu et al., 2022; 2024a). Eye-position traces were first differentiated to obtain velocity estimates, and saccades were identified when gaze velocity exceeded a threshold defined relative to the median-based velocity in that trial (using a threshold of five times the median; as we also used in de Vries and van Ede, 2024; Liu et al., 2024a;b; Wang and van Ede, 2025). To focus our analysis on saccades related to internal shifts of attention, we focused on fixational saccades that moved gaze away from the fixation dot (as in Liu et al., 2024a) and in the direction of the memorized location of the cued memory item (i.e., “toward” saccades). To understand whether and how our far/near manipulation affected the size of fixational saccades, we calculated the density of saccades as a function of saccade size, in a range spanning from 0 to 2° visual angle (using a sliding window with a step size of 0.05° and a width of 0.5°). Note that, because microsaccade biases and gaze-position biases index different aspects of gaze behavior, they were analyzed in distinct predefined time windows that were fully consistent with previous work studying these respective measures. While gaze-position biases were quantified in the established 400–1,000 ms window following cue onset (as first defined as the relevant window in van Ede et al., 2019), fixational saccades were analyzed in the established 200–600 ms window (as first defined as the relevant window in Liu et al., 2022; and later also used in Liu et al., 2024a;b). To maximize sensitivity, we again pooled the data from Experiments 2 and 3 for this additional analysis.

### Statistical analysis

To evaluate and compare gaze towardness time courses, we employed a cluster-based permutation approach (Maris and Oostenveld, 2007), which is well suited for evaluating multiple neighboring time points while avoiding multiple comparisons. In this approach, we generated a permutation distribution by randomly permuting trial-average time courses at the participant level 10,000 times and identifying the largest clusters after each permutation. *p* values were computed as the proportion of permutations where the largest postpermutation cluster exceeded the size of the observed cluster(s) in the original (nonpermuted) data. Using FieldTrip, we performed this permutation analysis with default cluster settings: grouping similarly signed significant data points from a univariate *t* test at a two-sided 0.05 alpha level and defining cluster size as the sum of all *t* values within the cluster.

Because our central question regarded spatial scaffolding for working memory, we focused on the spatial gaze bias as our primary outcome variable because this is a direct spatial index. For completeness, we also considered our behavioral performance data as a function of our conditions. We note however that performance data in the current study mainly served to ensure that participants were able to complete the task across our conditions. We had no specific hypotheses about these data. The gaze bias over the extracted a priori defined time window (based on van Ede et al., 2019) and the behavioral performance data (the accuracy and reaction times) were statistically evaluated using a two-way repeated-measures ANOVA with the factors whether the cued item was near or far and whether direction was sufficient (and distance-redundant) or direction was insufficient (and distance-useful) as a scaffold for separating the four items in working memory. ANOVA results were supplemented with post hoc *t* tests. As measures of effect size, we used partial eta squared for ANOVA and Cohen's d for follow-up *t* tests. *p* values of follow-up *t* tests were Bonferroni corrected for multiple comparisons. When we observed no difference between conditions, we additionally employed Bayesian statistics in Jamovi (The jamovi project, 2021; version 1.6) with default priors (*r* = 0.707). This allowed us to quantify evidence in favor of the null hypothesis (BF01).

### Data availability

The raw eye tracking and behavioral data are publicly available on OSF (https://osf.io/yras2/overview?view_only=a6575fceb5f348829b46c1fb8a81033a). 

### Code availability

Relevant codes and the processed data used to generate the figures are publicly available on OSF (https://osf.io/yras2/overview?view_only=a6575fceb5f348829b46c1fb8a81033a).

## Results

Human participants performed working-memory tasks in which we cued (via a central color retro-cue) the selection of one of four spatially separated visual items for an orientation comparison to the ensuing test stimulus (Fig. 1*a*). We presented items either near (3°) or far (6°) from the central fixation dot and used spatial biases in fixational gaze behavior during mnemonic selection as an implicit read-out of the spatial scaffold used for visual working memory (an approach that we applied successfully in several recent complementary studies: van Ede et al., 2019; Draschkow et al., 2022; Chawoush et al., 2023; de Vries et al., 2023; Liu et al., 2024a; Wang and van Ede, 2025).

In what follows, we first establish that our spatial marker can track the use of both “direction” and “distance,” when direction and distance are both useful as a spatial scaffold for individuating the four visual memory items—even when direction and distance were never asked about. We then show across two additional experiments that participants resort to a sparser spatial scaffold that abstracts away over distance, when direction alone is sufficient for individuating the four memory items.

The key outcome variable in our study was gaze during mnemonic selection. Before turning to our gaze data, we first confirmed that participants were able to perform this task well above chance in all three versions that we ran (Supplementary Fig. 1). Further note how we return to our performance data after delineating our primary gaze findings.

### Fixational gaze behavior during mnemonic selection tracks the use of both direction and distance as spatial scaffolding features for visual working memory

Figure 1*b* shows gaze time courses after retro-cues that prompted the selection of memory items that had been encoded left or right from fixation, at either the near (Fig. 1*b*, left) or far (Fig. 1b, right) position. Consistent with prior studies from the lab, we observed clear spatial biases in gaze in the direction of the original location of the cued memory item. Importantly, this occurred even though (1) there was nothing to look at to the left and right after the retro-cue, (2) our color retro-cue and ensuing test stimulus were presented centrally, and (3) we never asked participants about the original location of memory items.

To study whether distance was incorporated in the spatial scaffold used for working memory—in addition to direction—we collapsed left and right trials into a single measure of “towardness” (as in van Ede et al., 2019; 2020; Chawoush et al., 2023; de Vries et al., 2023) and overlayed towardness between trials with cues to near and far items. As shown in Figure 1*c*, we found that gaze after the retro-cue became significantly biased in the direction of the cued memory item, both when the cued item was near (cluster *p* < 0.001) or far (cluster *p* < 0.001). In addition, the spatial bias in gaze was clearly modulated by the memory item's distance at encoding, with a larger bias when cued to select the further item (cluster *p* < 0.001; Fig. 1*c*, black horizontal line).

The observed gaze biases originated from biases in fixational gaze behavior (as in Hafed and Clark, 2002; Engbert and Kliegl, 2003; van Ede et al., 2019; 2020)—and not looking back at the original location of the memorized item (as in Spivey and Geng, 2001; Ferreira et al., 2008; Johansson and Johansson, 2014; Bone et al., 2019; Wynn et al., 2019) at 3 or 6°, respectively. This can be appreciated by the heat maps (two-dimensional density plots) of the difference in gaze position following cues to select the left versus right items, as depicted in Figure 1*d* (see Supplementary Fig. 2 for the heat maps of gaze density following left and right cues separately).

These spatial biases in fixational gaze behavior during mnemonic selection confirm the use of space as a scaffold for mnemonic individuation, even if item locations are never asked about. These data further show that our gaze marker of this spatial scaffold is able, in principle, to track the use of both direction (left/right) and distance (near/far).

### Fixational gaze behavior reveals a sparse spatial scaffold that abstracts over distance when direction alone is sufficient for mnemonic individuation

In Experiment 1, there were always two memory items in each direction (two left and two right). Accordingly, direction (left/right) alone was insufficient as a spatial scaffold for individuating the four items, rendering distance useful as an additional scaffolding feature. Following this logic, we refer to this condition as “direction-insufficient → distance-useful” (Fig. 2*a*, left).

In Experiment 2, we included an additional condition in which each memory item was placed along a separate direction from fixation (up, down, left, and right), while again manipulating item distance. Critically, in this condition direction became sufficient (in principle) as a spatial scaffold for individuating the four memory items, potentially rendering distance redundant as an additional scaffolding feature. Accordingly, we refer to this condition as “direction-sufficient → distance-redundant” (Fig. 2*a*, right).

When considering the condition in which distance was useful as an additional scaffolding feature because direction alone was insufficient (Fig. 2*b*), we replicated our findings from Experiment 1. We again found that gaze after the retro-cue became significantly biased in the direction of the cued memory item, both when the cued item was near (cluster *p* < 0.001) or far (cluster *p* < 0.001) and found that the spatial gaze bias was again clearly modulated by the memory item's distance at encoding (Fig. 2*b*), with a larger bias when cued to select the further item (cluster *p* < 0.001).

Our key insight comes from the same spatial gaze bias in the condition in which all four memory items each had a unique direction (left, right, top, bottom). Here, we still observed clear gaze biases in the direction of the cued memory item (Fig. 2*c*), both when the cued item was near (cluster *p* < 0.001) or far (cluster *p* < 0.001). Critically, however, in this condition, we no longer found a modulation by distance (Fig. 2*c*). Importantly, this was observed even though we had presented the items at the exact same distances (3 and 6°) as in the alternative condition. This attenuated modulation by distance exclusively in this condition is consistent with the notion that, in this condition, direction was sufficient as a scaffold, affording working memory to “abstract away” over distance.

To further quantify this key finding, we collapsed these gaze biases over the predefined window of 400–1,000 ms after cue onset—a window chosen a priori based on van Ede et al. (2019). This aggregate gaze-bias measure is depicted in Figure 2*d*. Analysis of variance confirmed a statistically significant interaction between the factors “whether the cued item was near or far” and “whether direction was insufficient (distance useful) or sufficient (distance redundant)” (*F*_(1,24)_ = 9.13, *p* = 0.006, partial *η*^2^ = 0.276). Post hoc analysis confirmed that when direction was insufficient as a spatial scaffold for individuating all four memory items, participants had a larger gaze bias when selecting the far (*M* = 0.125 ± 0.028 [mean ± SE]) compared with the near (*M* = 0.065 ± 0.014) memory item (*t*_(24)_ = −2.872, *p*_Bonferroni_ = 0.05, Cohen's d = −0.574). However, when direction was sufficient for individuating the four items (because each memory item appeared in a distinct direction from fixation at encoding), participants showed highly similar gaze bias regardless of whether we cued the near (*M* = 0.093 ± 0.019) or the far (*M* = 0.097 ± 0.026) memory item (*t*_(24)_ = −0.401, *p*_Bonferroni_ = 1, Cohen's *d* = 0.08). A Bayesian analysis confirmed moderate evidence in favor of the null effect of no difference between far and near in the direction-sufficient condition (BF01 = 4.41). The key interaction pattern of interest—larger distance coding in the direction-insufficient compared with the direction-sufficient condition—was present in the majority of participants (Fig. 2*e*). Together, these data suggest the use of a “sparser” spatial scaffold in the direction-sufficient condition, in which item distance was redundant as an additional scaffolding feature.

Gaze heat maps, here split for trials with horizontal and vertical configurations, again revealed the fixational nature of these gaze biases (Fig. 2*f*,g).

### Our findings are driven by spatial scaffolding demands, not the mere presence of near and far items

In Experiment 2, we manipulated whether direction was sufficient (distance redundant) or insufficient (distance useful) as a spatial scaffold for individuating the four memory items by presenting items either in unique directions from fixation, or by presenting multiple items in the same direction from fixation. However, in the former condition, the four items were always all near or all far, while in the latter condition displays always contained two near and two far items. Accordingly, it remains possible that our findings of a distinct spatial scaffold between the two conditions reflects the mere presence of both near and far items in one condition, and only near or far items in the other condition.

To rule out this possibility, we designed Experiment 3 where we repeated our key manipulation, but this time ensured that displays always contained two near and two far items in both conditions (Fig. 3*a*). The direction-insufficient (distance-useful) condition mirrored Experiment 2 (Fig. 3*a*, left), while the direction-sufficient (distance-redundant) condition now always had two items placed at the “near” positions on one axis and another two items placed at the “far” positions on the orthogonal axis (Fig. 3*a*, right). Accordingly, both conditions now contained items at both distances, while direction was still sufficient (and distance redundant) as a spatial scaffold in one condition but not in the other.

Gaze data in Experiment 3 replicated those from Experiment 2: again, showing a larger fixational gaze bias when selecting the further item, that was only significant in the condition where direction was insufficient—and hence distance useful—for individuating the four memory items (Fig. 3*b*; cluster *p* < 0.001) and did not reach significance in the direction-sufficient condition in which distance was redundant (Fig. 3*c*; no far-vs-near cluster). These findings were again corroborated by a significant interaction (Fig. 3*d*; *F*_(1,24)_ = 7.54, *p* = 0.011, partial *η*^2^ = 0.239), with post hoc analysis confirming larger fixational gaze bias when selecting the far (*M* = 0.219 ± 0.039) versus near (*M* = 0.146 ± 0.025) item when direction was insufficient (distance useful) for item individuation (*t*_(24)_ = −3.518, *p*_Bonferroni_ = 0.011, Cohen's *d* = −0.704), but no significant difference between far (*M* = 0.158 ± 0.035) and near (*M* = 0.15 ± 0.028) when direction was sufficient and distance was redundant as a scaffolding feature (*t*_(24)_ = −0.55, *p*_Bonferroni_ = 1, Cohen's *d* = −0.11). A Bayesian analysis again confirmed moderate evidence in favor of the null effect of no difference between far and near in the direction-sufficient condition (BF01 = 4.13). We again note how this pattern was present in the majority of participants (Fig. 3*e*). The heat maps in trials with horizontal and vertical configurations again revealed the fixational nature of these gaze biases (Fig. 3*f*,g).

### Our findings are not due to conditional differences in performance

Having presented and replicated our primary findings across Experiments 2 and 3, we finally turn to two additional pieces of evidence that corroborate our conclusions.

**Figure 4.**
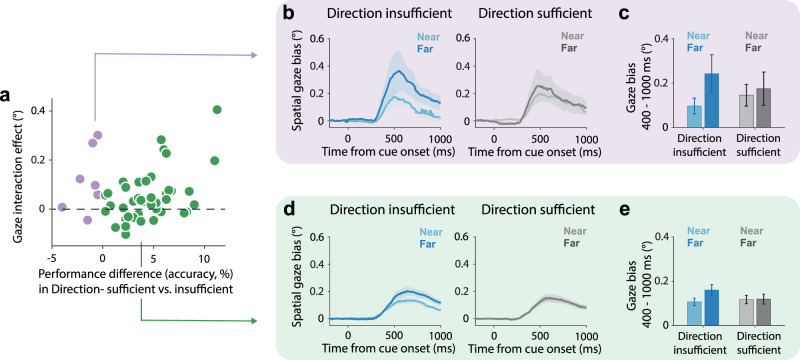
Differences in performance between our direction-sufficient and direction-insufficient conditions cannot explain the differences in distance-dependent coding as quantified but the interaction effect in the gaze-bias. ***a***, Scatterplot showing each participant's difference in accuracy between direction-sufficient versus direction-insufficient conditions (*x*-axis), plotted against their interaction in gaze effect (i.e., the difference in the far–near bias between direction-insufficient and direction-sufficient conditions; *y*-axis). The nonsignificant correlation (*r* = 0.21, *p* = 0.15, BF01 = 2.08) suggests that the size of the gaze interaction effect is unrelated to differences in accuracy (and by extension differences in difficulty or crowding) between our direction-sufficient and direction-insufficient conditions. ***b***, ***c***, Gaze-bias patterns across conditions for the minority of participants who were better (higher accuracy) in the direction-insufficient condition. Note how these additional visualizations serve solely to better understand the data in panel ***a***. Because there were only seven participants in this subgroup, no formal statistical tests were conducted on the visualized data for this group, nor for the comparison between groups. ***d***, ***e***, Gaze-bias patterns across conditions for the majority of participants who were worse (lower accuracy) in the direction-insufficient condition.

First, we observed a significantly larger effect of item distance in the direction-insufficient condition. So far, we interpreted this from the perspective that only in this condition distance was useful as an additional spatial feature aiding item individuation. However, besides the “sufficiency” of directional information for item individuation, our conditions also differed in task performance, with the direction-insufficient condition being, on average, more difficult and participants achieving slightly lower accuracy. In Experiment 2, the accuracy in the direction-insufficient condition (*M* = 0.74 ± 0.02) was lower than in the direction-sufficient condition (*M* = 0.77 ± 0.02; *t*_(24)_ = −5.39, *p* < 0.001, Cohen's *d* = −1.08). Likewise, in Experiment 3, the accuracy in the direction-insufficient condition (*M* = 0.79 ± 0.01) was lower than in the direction-sufficient condition (*M* = 0.82 ± 0.01: *t*_(24)_ = −5.64, *p* < 0.001, Cohen's *d* = −1.13). Could such differences in performance—as an observable measure of putative differences in difficulty and potential differences in perceived crowding—explain our findings? To assess this, we analyzed our key gaze patterns as a function of the observed difference in task accuracy between these conditions (Fig. 4). Critically, we found no significant correlation (*r* = 0.21, *p* = 0.15, BF01 = 2.08) between our main gaze-bias interaction effect and the individual-specific difference in accuracy between these conditions (Fig. 4*a*). To appreciate this, when visualizing the data separately for participants who actually showed better (not worse) performance in the direction-insufficient condition, we found that the overall pattern of the gaze bias across our four conditions was similar (Fig. 4*b*,*c*), consistent with the lack of any significant correlation. Note that because the subgroup with the more rare negative performance difference had only seven participants, no formal statistical tests were conducted for this subgroup, nor for the comparison between subgroups. Rather, to evaluate the (lack of a) relation, we used the continuous data across all 50 participants that we evaluated by means of the correlation analysis presented above (Fig. 4*a*).

These data argue against differences in performance, and thereby potential differences in difficulty or perceived crowding, as alternative explanations for our findings.

By pooling the data between Experiments 2 and 3, we also increased sensitivity for analyzing our gaze data in a complementary manner by identifying individual fixational saccades (microsaccades) in an analysis window inspired by prior studies [first used in Liu et al. (2022) and later also in Liu et al. (2024a,b)] and analyzing these as a function of saccade size. For this we focused on fixational saccades toward the memorized location of the cued memory item, as these are the saccades that have been shown to reflect internal attention shifts (Liu et al., 2022) and that contribute to the gaze-position bias “toward” the cued memory item. We note up front that the outcomes of these more challenging analyses (for which we had a relatively scarce amount of data available) were inevitably more tentative than the gaze-position biases reported so far. Even so, these analyses, presented in Supplementary Figure 3, suggested that the reported gaze-position findings are paralleled by a modulation in the size of fixational saccades, up to 2° (well below the distance of the far item at 6°, consistent with the heat maps in preceding Figs. 1–3). Specifically, just like for our primary findings, we found an increase in the size of fixational saccades when selecting the far item, but only when direction alone was insufficient for item individuation (Supplementary Fig. 3; cluster *p* < 0.05).

## Discussion

We unveil how humans engage fundamentally distinct spatial codes for retaining visual representations in working memory, depending on the utility of spatial features as a scaffold for memory. Leveraging spatial biases in fixational gaze behavior during mnemonic selection as an implicit read-out of spatial scaffolding for visual working memory (as in Draschkow et al., 2022; Chawoush et al., 2023; de Vries et al., 2023; Liu et al., 2024a), our present data unveil the phenomenon of “sparse spatial scaffolding” for visual working memory, whereby working memory flexibly resorts to using the minimal spatial features necessary for the separation and selection of individual memory contents.

Rather than investigating how we memorize space (cf. Funahashi et al., 1989; Awh et al., 1998; Postle and D’Esposito, 2003; Sprague et al., 2014; Foster et al., 2016; Aagten-Murphy and Bays, 2019; Kwak and Curtis, 2022), we asked how we use space for memorizing. We never asked participants to report memorized item direction nor distance. Instead, space served as an organizing medium (scaffold) serving the separation and selection of individual memory contents. While ample studies have made clear that space is a useful scaffold for working memory (Griffin and Nobre, 2003; Treisman and Zhang, 2006; Hollingworth, 2007; Kuo et al., 2009; Theeuwes et al., 2011; Abrahamse et al., 2014; Pertzov and Husain, 2014; Manohar et al., 2017; Schneegans and Bays, 2017), how space is used has remained elusive. Inspired by several findings (Brady et al., 2009; Lee et al., 2013; Kwak and Curtis, 2022), we hypothesized that working memory may abstract away from the veridical spatial layout at encoding to a more efficient or “sparser” spatial scaffold—as long as it serves the job of mnemonic individuation. We found evidence for this. In our setup, this was achieved by abstracting away over distance when direction was sufficient for mnemonic individuation, but incorporating distance as an additional scaffolding feature when direction was insufficient (when multiple items existed along the same direction). It is worth noting that an even sparser representation may have retained no spatial information whatsoever: after all, we never asked participants about item location. However, this is not what we found. Rather, our findings build on ample prior studies that have shown how space serves an organizing (scaffolding) role in working memory. Our findings extend prior work by showing that such a spatial scaffold can be flexibly adjusted to a sparser format when a sparser format suffices item individuation.

Ultimately, the strength of our inference hinges on the suitability of our spatial marker for studying spatial scaffolding for working memory. Our findings build directly on several prior studies where we successfully leveraged spatial biases in fixational gaze behavior to study spatial scaffolding for working memory—without ever asking participants about memorized item location (van Ede et al., 2019; Draschkow et al., 2022; Chawoush et al., 2023; de Vries et al., 2023; de Vries and van Ede, 2024; Liu et al., 2024a; Wang and van Ede, 2025). Here, we for the first time use this implicit marker to study the use of distance. In all three experiments, we confirm that this marker—while fixational in nature—is sensitive to memorized item distance, in principle. Our key findings of an attenuated “distance code” when distance is a redundant scaffolding feature suggests the use of a sparse spatial scaffold in which distance can be abstracted away. Note, though, that we do not claim that there is zero distance coding in the direction-sufficient (distance-redundant) condition where we could not establish a significant effect of item distance. This null effect may also reflect insufficient sensitivity of our marker. Crucially, however, this same marker showed a significantly larger distance effect when distance was useful (versus redundant) as a spatial scaffolding feature. Our central conclusion thus involves a comparatively sparser code when distance was redundant, supported by a significant interaction.

At the same time, the observed sensitivity to distance (when useful) reveals how biases in fixational gaze behavior signal more than a low-level “orienting response” that merely reflects in which direction to orient—as may be predicted considering this gaze bias may reflect a spillover from activity in subcortical brain circuitry with evolutionary roots in orienting (cf. Corneil and Munoz, 2014; Cisek, 2019; Zhou et al., 2023). While remaining fixational in nature, our data reveal how this gaze bias is sensitive to “high-level” cognitive coding by tracking the flexible utilization of distinct spatial scaffolds for working memory that can either incorporate or abstract away over distance. This adds to growing appreciation of higher-level cognitive processing within deep-brain structures, such as the superior colliculus (Müller et al., 2005; Knudsen, 2011; Krauzlis et al., 2013; Liu and van Ede, 2024) that has been implicated in spatial biases in fixational gaze behavior that we report here (Hafed et al., 2009).

By studying spatial coding for working memory following a cue that directed internal selective attention to a specific memory item, our findings complement prior studies on selective attention that also varied item distance (Hafed and Clark, 2002; Busch et al., 2004; Bahramisharif et al., 2011; Schaffer et al., 2011; Bressler et al., 2013; Roijendijk et al., 2013; Papaioannou and Luck, 2020; Wang et al., 2021; Gayet et al., 2024), even if these typically studied externally directed attention. Interestingly, at least several studies reported a relative invariance of spatial modulations in neural activity (Roijendijk et al., 2013; Papaioannou and Luck, 2020; Wang et al., 2021) or fixational gaze behavior (Hafed and Clark, 2002) to the attended target's distance, while other studies did report distance-dependent modulations (Busch et al., 2004; Bahramisharif et al., 2011; Schaffer et al., 2011; Bressler et al., 2013). We unveil a key variable that may underpin whether or not this is found: the utility of distance for the task at hand (see also Gayet et al., 2024).

Though our starting point was different, our experimental manipulations resemble those in complementary research on the role of eccentricity and crowding in vision (Deubel and Schneider, 1996; Carrasco and Frieder, 1997; Intriligator and Cavanagh, 2001; Carrasco et al., 2003; Strasburger et al., 2011; Harrison et al., 2013; Staugaard et al., 2016; Kewan-Khalayly and Yashar, 2022; van Heusden et al., 2023) and visual working memory (Tamber-Rosenau et al., 2015; Harrison and Bays, 2018; Yörük et al., 2020). This literature has typically focused on other questions, such as how visual resolution changes when moving from central to peripheral vision. Our task was not designed to tap into such differences. We used clearly visible stimuli and placed them 3° apart. Also, when considering performance, we did not observe lower accuracy for the far item in any of the three experiments (Supplementary Fig. 1). Instead, we here focused on whether and how distance was used as a scaffold for visual working memory when distance is a useful or a redundant scaffolding feature (in addition to direction). While our direction-insufficient (distance-useful) condition may invoke more crowding, we note that our key findings do not consist of a general increase or decrease of the fixational gaze bias in this condition (but rather an interaction with distance). Furthermore, the main insight from our study—that distance is abstracted away when it is a redundant scaffolding feature—originated from our condition with minimal crowding, when all items were presented in a distinct direction. Finally, we note how our gaze interaction effect was uncorrelated with accuracy differences between our direction-insufficient and direction-sufficient conditions and was observed even in those participants whose performance was better (not worse) in the direction-insufficient condition (Fig. 4). These findings suggest that differences in performance (as an observable measure of putative differences in difficulty, or potential differences in perceived crowding) between our experimental conditions do not explain our main gaze results.

While overall differences in difficulty thus unlikely explain our key gaze-bias findings, we cannot rule out that participants may have adopted different mnemonic strategies across conditions—in so far as these strategies did not lead to differences in accuracy. For example, it is conceivable that in our condition where all items had a unique direction, participants may have chunked/grouped the items differently. Such an account is not necessarily inconsistent with our interpretation. Moreover, the differential use of distance between conditions can also be thought of as a difference in “strategy.” Future studies are required to delineate to what extent such differences may be contingent on specific task parameters.

Having reported the phenomenon of sparse spatial scaffolding for visual working memory, our data open relevant avenues for future research. Our data leave unaddressed how the spatial “pruning” of visual working memory develops between encoding and our moment of exposing the spatial scaffold after the cue and how such spatial pruning occurs across the multitude of brain areas involved in working memory (Christophel et al., 2017). Our findings hint most directly at the spatial scaffold used by oculomotor circuitry in the brain, of which our gaze marker is a peripheral fingerprint (Hafed et al., 2009). Developing ways to continuously track the transformations in the spatial scaffold for working memory across time and across the brain remains challenging but also exciting avenues awaiting future research.
